# Targeting the Heterogeneous Genomic Landscape in Triple-Negative Breast Cancer through Inhibitors of the Transcriptional Machinery

**DOI:** 10.3390/cancers14184353

**Published:** 2022-09-07

**Authors:** Vera E. van der Noord, Bob van de Water, Sylvia E. Le Dévédec

**Affiliations:** Division of Drug Discovery and Safety, Leiden Academic Centre for Drug Research, Leiden University, 2333 CC Leiden, The Netherlands

**Keywords:** triple-negative breast cancer, transcriptional machinery, transcription-associated cyclin-dependent kinases (CDKs), bromodomain and extra-terminal (BET) proteins

## Abstract

**Simple Summary:**

Triple-negative breast cancer (TNBC) is the most aggressive subtype of breast cancer with limited options for therapy. Cancer development and maintenance is dependent on the production of oncogenic proteins. In TNBC, the production of many of these proteins is changed, and therapeutic targeting of one of these proteins is often not effective. However, a common step in the production of these proteins, transcription, can be effectively targeted through inhibition of the transcriptional machinery. These inhibitors can specifically suppress the production of oncogenic proteins important for TNBC. At the same time, the altered production of these proteins might interfere with the sensitivity of these cancer cells to these inhibitors. This review provides an overview of the effects of inhibitors of the transcriptional machinery on the abnormal oncogenic protein production in TNBC. This overview thereby highlights the research further needed in this field, and the potential opportunities of using these inhibitors for TNBC.

**Abstract:**

Triple-negative breast cancer (TNBC) is an aggressive subtype of breast cancer defined by lack of the estrogen, progesterone and human epidermal growth factor receptor 2. Although TNBC tumors contain a wide variety of oncogenic mutations and copy number alterations, the direct targeting of these alterations has failed to substantially improve therapeutic efficacy. This efficacy is strongly limited by interpatient and intratumor heterogeneity, and thereby a lack in uniformity of targetable drivers. Most of these genetic abnormalities eventually drive specific transcriptional programs, which may be a general underlying vulnerability. Currently, there are multiple selective inhibitors, which target the transcriptional machinery through transcriptional cyclin-dependent kinases (CDKs) 7, 8, 9, 12 and 13 and bromodomain extra-terminal motif (BET) proteins, including BRD4. In this review, we discuss how inhibitors of the transcriptional machinery can effectively target genetic abnormalities in TNBC, and how these abnormalities can influence sensitivity to these inhibitors. These inhibitors target the genomic landscape in TNBC by specifically suppressing MYC-driven transcription, inducing further DNA damage, improving anti-cancer immunity, and preventing drug resistance against MAPK and PI3K-targeted therapies. Because the transcriptional machinery enables transcription and propagation of multiple cancer drivers, it may be a promising target for (combination) treatment, especially of heterogeneous malignancies, including TNBC.

## 1. Introduction

Breast cancer is the most common type of cancer in women and affects approximately 1 in 8 women. Triple-negative breast cancer (TNBC) is one of the most aggressive subtypes of breast cancer and accounts for approximately 15% of all breast cancer cases [1]. TNBC is defined by the lack of estrogen (ER), progesterone (PR) and human epidermal growth factor receptor 2 (HER2) expression, and therapies against these receptors are therefore not effective in TNBC patients. Although a subset of TNBC patients respond relatively well to neo-adjuvant chemotherapy and surgery, most of these patients still experience early recurrence and have poor responses to adjuvant chemotherapy, while no other effective alternatives are available [2,3]. Targeted therapies, including PARP inhibitor and immune checkpoint blockade therapy, have recently been approved for the treatment of a subset of TNBC patients, yet this is not applicable to all patients and most treated patients still have poor clinical prognosis due to an eventual relapse. Therefore, more effective (combination) treatments targeted against TNBC drivers are urgently needed.

**Figure 1:**
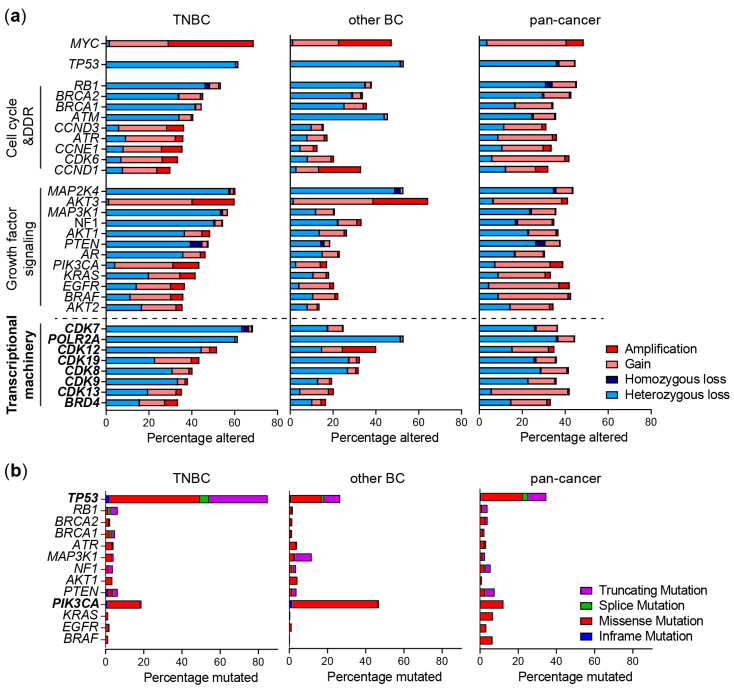
Genomic alterations of frequently altered pathways and the transcriptional machinery (TM) in TNBC. (**a**) Copy number alterations and (**b**) mutations in genes from TNBC patients from the METABRIC study (TNBC and other BC) or TCGA (pan-cancer) derived from cBioportal. Shown are the most frequently altered genes within cell cycle, DNA damage response (DDR) and growth factor signaling pathways.

The main problem of finding targeted therapy for TNBC is that its actionable drivers are very heterogeneous among patients and within one tumor. Through their transcriptomic profile, TNBC patients can be classified in different molecular subtypes, including the basal-like 1 and 2, immunomodulatory, mesenchymal-like, mesenchymal stem-like and luminal AR subtypes [4]. In addition, genomic profiling has identified numerous mutations and copy number alterations in TNBC [5,6,7,8,9]. However, these alterations are also very heterogenous among patients, except for *TP53*, which is compromised in most TNBC patients, but cannot be well exploited with therapy. Other frequent genomic alterations include mostly mutations or copy number alterations in DNA damage signaling and repair pathways (e.g., *BRCA1*), cell cycle regulators (e.g., *RB1*, *CDK6*, *CCND1*), growth factor signaling pathways (e.g., *PIK3CA*, *EGFR*) or their downstream transcription factors (e.g., *MYC*) (Figure 1a,b) [5,6,9]. Another great challenge in treating TNBC is the large intratumor heterogeneity, which is widely supported by recent single cell RNA and DNA sequencing data of TNBC primary tumors [10,11,12,13,14]. Within one tumor, different cell subpopulations can be assigned to different TNBC molecular subtypes. These subpopulations also have major differences in oncogenic driver mutations and copy number alterations [10,11,13]. This intratumor heterogeneity can contribute to drug resistance by providing cell subpopulations with differential therapeutic vulnerabilities [15,16]. Most targeted therapies that have been in clinical trials are directed against one, or two of the genetic alterations characterizing the bulk tumor. Although targeted therapies have provided benefits for more uniformly driven cancers, such as *BRAF*-driven melanoma or hormone-positive breast cancer, the heterogeneity in TNBC limits clinical responses to most targeted therapies. A promising strategy to combat TNBC would require the simultaneous tackling of multiple alterations in the complex genomic landscape of this cancer. Many of these abnormalities eventually drive cancer progression through selectively driving oncogenic transcriptional programs. This transcriptional addiction is a common feature of most cancers [17], and the transcriptional machinery (TM) is thus a common hub that may be targeted to simultaneously tackle multiple TNBC drivers.

**Figure 2:**
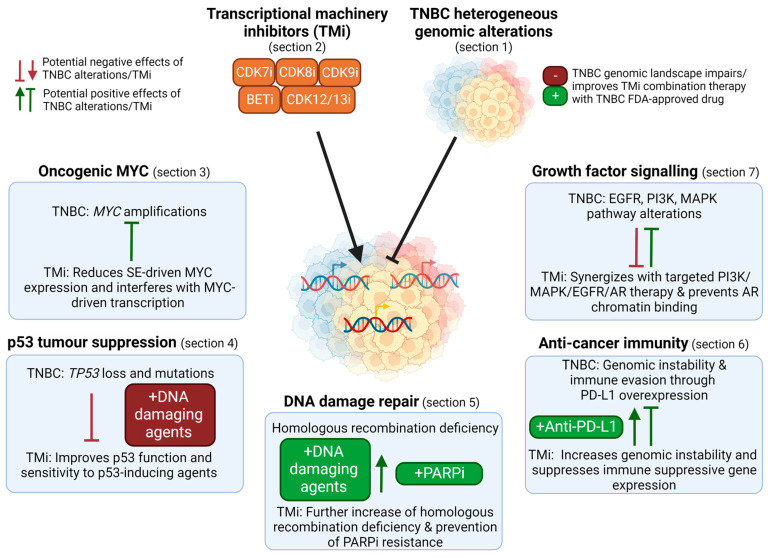
Overview of the use of TM inhibitors (TMi’s) to tackle the genomic landscape in TNBC. TMi’s can suppress super-enhancer (SE) driven MYC expression and interfere with MYC-driven transcription (Section 3). As TMi’s can improve p53 function and thereby synergize with DNA damaging agents, genomic loss of *TP53* in TNBC can reduce the efficacy of these combination therapies (Section 4). TNBC is characterized by frequent homologous recombination deficiencies and TMi’s further induce genomic instability, thereby sensitizing cells to DNA damaging agents and PARP inhibitors. Despite their genomic instability that could evoke immune responses, TNBCs suppress this, for example through PD-L1 overexpression (Section 6). TMi’s further induce genomic instability and prevent immune suppressive gene expression, improving anti-cancer immunity and anti-PD-L1 therapy. Furthermore, TMi’s can improve therapies directed against the elevated levels of growth factor or AR signaling pathways in TNBC, such as PI3K and MEK inhibitors (Section 7). This figure was created with BioRender.com (accessed on 8 August 2022).

Several components of the TM can be effectively and selectively targeted, such as transcriptional cyclin-dependent kinases (CDK) and bromodomain and extra-terminal (BET) proteins that induce transcription by regulating RNA polymerase II activity. Understanding how these TM inhibitors (TMi’s) interact with various cancer drivers is critical to rationally design therapeutic strategies, for example, biomarker-based patient selection and combination therapies. In this review, we discuss: (1) how the TM can be targeted; (2) how its activity is altered in TNBC; and, (3) how it interferes with the heterogeneous TNBC drivers. Overall, this review identifies the opportunities, challenges and knowledge gaps concerning the genomic landscape of TNBC and its indirect targeting by TMi’s (Figure 2).

## 2. Targeting the TM in TNBC

Transcription of mRNA occurs through a series of steps, including transcription initiation, pausing, elongation and termination, which are driven by RNA polymerase II. RNA polymerase II is regulated by a complex set of proteins composing the TM, which has recently been extensively reviewed [18]. Within the TM, only a limited number of proteins can currently be pharmacologically and selectively inhibited to halt uncontrolled transcription. In this review we focus on these actionable proteins, which mainly include the transcriptional CDKs 7, 8, 9, 12, 13 and 19, and the bromodomain and extra-terminal (BET) protein bromodomain-containing protein 4 (BRD4).

### 2.1. Regulation of Transcription by the TM

CDK7 regulates transcription initiation and promoter escape. Before transcription is initiated, a large complex of general transcription factors, the pre-initiation complex (PIC), is recruited to the gene promoter [18]. Some of these general transcription factors can recognize specific DNA motifs and can subsequently recruit other factors from the PIC. The PIC consists of multiple smaller complexes, including, but not limited to, TFIIH, RNA polymerase II and Mediator. CDK7, part of the TFIIH complex and dependent on MAT1 and cyclin H, phosphorylates serine 5/7 residues of the C-terminal domain (CTD) of RNA polymerase II, which stimulates further conformation changes and co-factor binding to initiate transcription. Transcription initiation is also mediated through another part of PIC, the Mediator complex, of which the kinase module includes CDK8, and its paralog CDK19, and Cyclin C. This complex mediates interactions between RNA polymerase II and transcription factors. CDK8 further regulates these interactions by phosphorylating these transcription factors and altering Mediator conformation, which either promotes or inhibits transcriptional activity [19]. After transcription is initiated, DRB Sensitivity Inducing Factor (DSIF) and Negative Elongation Factor (NELF) bind to induce the pausing of RNA polymerase II [18].

CDK7 further regulates transcription by phosphorylating CDK9. CDK9 and cyclin T, together referred to as positive transcription elongation factor b (p-TEFb), are part of the super-elongation complex (SEC), which is required for transcription pause release and further elongation [18]. P-TEFb releases the TM from promoter-proximal pausing by phosphorylating NELF, DSIF and serine 2 residues of the CTD of RNA polymerase II. The activity and recruitment of p-TEFb is strongly regulated by the SEC itself, but also by BRD4, which is a histone-reader that accumulates on acetylated enhancer and promoter regions [20]. This histone acetylation and recruitment of BRD4 is regulated by transcription factors and histone modifiers. In turn, BRD4 can also recruit other transcription factors in addition to CDK9 and histone modifiers and chromatin remodelers to further enhance transcription [21,22]. In addition to BRD4, other BET proteins such as BRD2 and BRD3 also regulate transcription through similar, yet distinct, mechanisms [23]. Histone modifications thus provide a key layer of epigenetic control underlying the activity of the TM, not only by recruiting BET proteins, but also by altering chromatin structure [24]. For example, active enhancers are characterized by H3K27Ac and H3K4Me1 modifications, while silencer regions are characterized by H3K27Me3 modifications [25]. Histone modifying enzymes, such as histone acetyltransferases (HAT), histone deacetyl transferases (HDAC), histone lysine methyltransferases (KMT) or histone lysine demethylases (KDM) regulate these modifications [24]. Inhibitors of these enzymes have therefore also been evaluated as potential anti-cancer therapeutics. They may function through de-repression of silenced tumor suppressor genes (e.g., HDAC inhibitors) or repression of otherwise highly transcribed oncogenes.

Efficient elongation and subsequent RNA processing and termination are further facilitated by CDK12 and CDK13, both associated with cyclin K. CDK12 and CDK13 cooperatively contribute to phosphorylating serine 2 residues of the CTD of RNA polymerase II to promote transcription elongation [26]. In addition, CDK12 and CDK13 interact with various RNA processing and splicing factors, and their inhibition prevents the phosphorylation of several of these [27,28]. Some of these factors are involved in the polyadenylation of transcripts, and CDK12 depletion especially disrupts the expression of long genes by inducing pre-mature cleavage and intronic polyadenylation of the transcripts [27,29,30]. Importantly, as will be discussed in this review, (partial) inhibition of the various transcription-associated CDKs and BRD4 does not affect global transcription, but rather results into gene-specific effects that can be exploited for cancer therapy (Figure 2 and Table 1).

### 2.2. Genomic Alterations of the TM

Compared to well-known tumor suppressor genes or oncogenes such as *TP53*, *MYC* and *PIK3CA*, genes expressing components of the TM are neither frequently mutated, nor have homozygous (affecting both alleles) amplifications or deletions in TNBC or other cancer types (Figure 1a,b) [8]. However, copy number gains or hemizygous losses (affecting one allele) are frequently identified, which often co-occur with well-known driver alterations. Especially hemizygous copy number losses of *CDK7* are more frequently observed in TNBC (72%) versus other cancers (27%) [29], which co-occurs with losses in DNA damage response genes in the 5q13 region, such as *RAD17* (Figure 1a).

Although mutations in most CDKs are rarely observed, *CDK12* mutations are observed in different cancer types (2.6% of all cases, enriched in ovarian and prostate cancer) which are often homozygous and lead to a loss function [29,30,31]. While these loss-of-function mutations are not common in TNBC, a large proportion of TNBC tumors express relatively low *CDK12* protein levels, and heterozygous *CDK12* copy number losses do occur relatively frequently (Figure 1a) [32]. In contrast, in HER2-positive breast cancer, the *CDK12* gene is often co-amplified in the *HER2* amplicon [8]. Although *BRD4* amplifications are enriched especially in ovarian cancer, they are also relatively frequently observed in (basal) breast cancer (Figure 1a) [33]. In addition, due to their genomic proximity, *POLR2A* (encoding RNA polymerase II subunit A) hemizygous losses frequently co-occur with *TP53* copy number losses [34]. Interestingly, these alterations may alter sensitivity to TMi’s, which may, in TNBC, be particularly exploited through reduced CDK7 and POLR2A expression.

### 2.3. Targeting the TM

Multiple selective and potent TMi’s have entered clinical trials (Table 1 and Table 2). In the past, most transcriptional CDK inhibitors were unspecific, inhibiting multiple CDKs with similar potency, such as the CDK inhibitors dinaciclib and flavopiridol. This lack in specificity limited clinical efficiency due to a narrow therapeutic window and limited the understanding of the function of individual CDKs. There are now several selective CDK7 (e.g., CT7001 and SY5609) and CDK9 (e.g., CYC065/fadraciclib and VIP152/BAY1251152) inhibitors in Phase I/II clinical trials for treatment of various hematological and solid malignancies, including TNBC (Table 2). Due to high similarity in kinase domain of CDK orthologues, there are no kinase inhibitors completely selective for CDK8, CDK19, CDK12 or CDK13 alone, but selective dual inhibition of CDK8/CDK19 and CDK12/CDK13 has been achieved. CDK12/13 can be inhibited through THZ531 and SR-4835, yet these inhibitors have not been clinically evaluated [35,36]. CDK8/19 inhibitors SEL120 and BCD-115 have recently entered clinical trials (Table 2). New opportunities for more selective targeting of these orthologues with highly similar kinase domains have been provided by the PROTAC technology, enabling the development of selective PROTAC inhibitors for CDK8 and CDK12 (Table 1) [37,38]. BET inhibitors (e.g., JQ1) often selectively inhibit BET family members over other proteins, but usually do not discriminate between BRD2, BRD3, and BRD4. Several PROTAC BET degraders have been developed, some of which are selective for BRD4 over other BET proteins, such as MZ1 [39]. BET inhibitors (mostly targeting BRD2, BRD3 and BRD4) have been tested in multiple clinical trials, however, these have not yet resulted in phase III studies due to treatment-related adverse events or limited efficacy (Table 2). Therefore, newer strategies to target BET proteins include more localized therapies using nanoparticles and prodrugs, more specific inhibition of one of the two bromodomains, and/or more specific inhibition of individual BET proteins (e.g., BRD4) [40,41,42,43]. Altogether, transcriptional CDKs and BET proteins are thus key targetable components of the TM, and, as discussed in the next sections, their inhibition can selectively disturb the transcription of multiple TNBC drivers.

## 3. TM Inhibition to Target MYC and Other Super-Enhancer Driven Oncogenes

**Figure 3:**
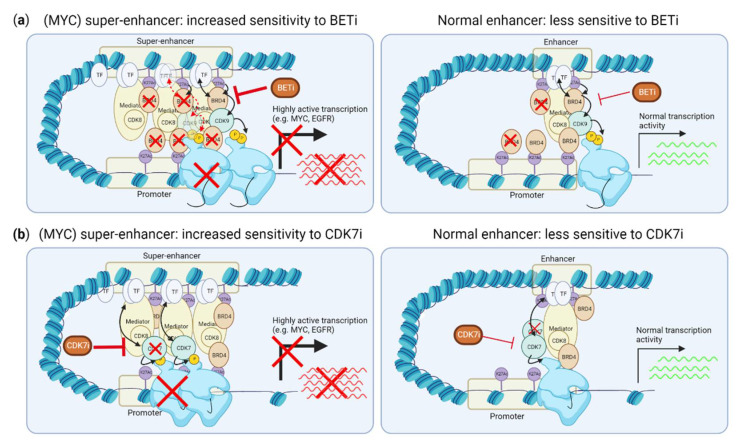
Effects of CDK7 and BET inhibition on super-enhancer driven transcription. (**a**) BRD4 is enriched at super-enhancers, and its inhibition preferentially disrupts this enrichment and expression driven by super-enhancers, not normal enhancers. (**b**) CDK7 inhibition preferentially reduces RNA polymerase II binding to the transcription start site of genes driven by super-enhancers. This figure was created with BioRender.com (accessed on 8 August 2022).

*MYC* is the most frequently amplified oncogene in TNBC (>60% have copy number gains or amplifications, Figure 1a) [8]. This oncogenic and pleiotropic transcription factor stimulates various processes important for tumor initiation, stemness, growth and drug resistance. MYC is therefore an attractive drug target, yet the development of drugs against it has been challenging [44]. Nevertheless, clinical trials with the first direct MYC inhibitors have recently started. Interestingly, TMi’s also interfere with MYC expression and function, and could therefore be used to target it indirectly (Figure 2 and Figure 3).

### 3.1. Inhibiting Super-Enhancer Induced Expression Suppresses MYC-Driven Transcription

TMi’s, including CDK7 and BET inhibitors, can specifically suppress transcription promoted by so-called “super-enhancers”, including the transcription of many (TNBC) oncogenic drivers, such as *MYC* (Figure 3a,b). Super-enhancers are genomic regions that contain multiple clusters of active enhancers. They are characterized by enriched H3K27Ac modifications and enriched binding of multiple transcription coactivators, such as MED1 and BRD4, which strongly drive transcription [45]. BRD4 binds to H3K27Ac on enhancers. While BRD4 thereby binds both normal enhancers and super-enhancers, super-enhancer driven transcription is more sensitive to BRD4 inhibition than transcription driven by normal enhancers (Figure 3a) [46,47,48,49]. While the exact mechanism of increased sensitivity of super-enhancers over normal enhancers is not fully understood, BRD4 inhibition more strongly reduces binding of BRD4 to super-enhancers than to normal enhancers [49]. As MYC overexpression is frequently caused by super-enhancer driven transcription, this super-enhancer suppression by BET inhibitors is therefore strongly associated with reduction of MYC expression and MYC-driven transcription. Accordingly, c-MYC- or N-MYC-driven cancer cells, including TNBC, are more sensitive to BRD4 inhibition [47,50,51,52]. However, as not all studies evaluated the presence of super-enhancers around the *MYC* or *NMYC* gene, it is unclear whether this selective inhibition of c-MYC or N-MYC is solely effective when MYC overexpression is driven by super-enhancers, or also when the *MYC* or *NMYC* genes themselves are amplified, without a gain in super-enhancer activity. Ectopic expression of MYC confers resistance to BRD4 inhibition, supporting the notion that BRD4 inhibition is mainly effective for inhibiting super-enhancer driven MYC expression [53]. Importantly, although evaluated in TNBC cell lines and a small number of TNBC tumors [48,54,55], the super-enhancer landscape in TNBC tumors, including the frequency of super-enhancer driven MYC expression, is yet to be defined.

Resistance to BET inhibitors is caused by re-expression of MYC via epigenetic plasticity of active (super) enhancers and induced transcription of *MYC* by β-catenin/Wnt signaling [56,57,58,59]. β-catenin signaling is often deregulated in TNBC, and thus inhibition of it may also be needed in combination with BET inhibition [57,60]. Of interest, CDK8 and CDK12 drive β-catenin/Wnt signaling and their inhibition thereby suppresses Wnt-driven MYC expression [61,62,63,64]. In contrast with BRD4, CDK8/19 inhibition activates super-enhancer activity, yet in leukemic cells this resulted in inhibition of proliferation by induction of super-enhancer driven transcription of tumor suppressor genes [65]. It is currently not fully understood if cancer cells may in general be sensitive to super-enhancer deregulation, both induction and suppression, or if either one of the two is more effective, depending on the (epi)genetic background of the cancer. In addition, CDK8/19 depletion increases the activation of BRD4-bound super-enhancers by redistributing MED12 chromatin occupancy, which sensitizes cells to BET inhibition [66].

Inhibition of CDK7 is associated with reduced occupancy of RNA polymerase II and suppression of transcription, which is more pronounced for super-enhancer driven genes, including TNBC oncogenes *MYC*, *EGFR* and *SOX9* (Figure 3b) [54,67,68]. Therefore, MYC-driven cancers, including TNBC, are more sensitive to CDK7 inhibition [54,67,68,69]. CDK7 inhibition also inhibits STAT3 phosphorylation, and reduces binding of STAT3 on the promoter of its targets, including *MYC* [70]. In addition to the *MYC* promoter, STAT3 binding motifs were also enriched on super-enhancers perturbed by CDK7 inhibition in TNBC [54]. STAT3 is frequently activated in TNBC and is associated with a worse prognosis [71]. Altogether, the disruption of super-enhancer driven transcription by CDK7, BRD4 and, potentially, CDK8 inhibition may thus target many TNBC driver genes at once, including *MYC*. Of note, while CDK7, CDK8 and BRD4 inhibition may interfere with the response of the TM to histone modifications, such as active enhancers, inhibitors of histone modifiers can alter the histone modifications themselves. Even HDAC inhibitors that inhibit deacetylation can, unexpectedly, reduce super-enhancer H3K27 acetylation and alter the (super-)enhancer landscape and BRD4 recruitment to gene bodies and enhancers [72,73,74].

### 3.2. CDK9 and Myc-Dependent Transcription Pause-Release and Gene Expression

CDK9 inhibition also represses N-MYC expression through binding to both super-enhancer and promoter regions of *MYCN* [75]. However, this super-enhancer regulation by CDK9 is less described and may not be applicable to the normal *MYC* gene. In contrast, CDK9 inhibition also causes induction of compensatory MYC expression via a BRD4-dependent recruitment of the remaining CDK9 from inactive p-TEFb [76]. This specifically promotes p-TEFb activation on the *MYC* promoter and underlies the synergy of CDK9 inhibition with BET inhibition. By suppressing MYC activity, cancer cells can also become resistant to chemotherapy [77]. CDK9 inhibition reverses this suppressed MYC activity, and thereby re-sensitizes cells to chemotherapy. MYC protein expression correlates with sensitivity to CDK9 inhibition, which is also increased upon ectopic MYC expression, suggesting this differential sensitivity is independent of super-enhancer driven expression [78].

While most transcription factors initiate transcription, MYC amplifies transcription by inducing pause release [79,80,81,82]. Mechanistically, MYC recruits CDK9 and SEC factors to the paused RNA polymerase II machinery. Moreover, MYC amplifies global transcription by antagonizing CDK9 sumoylation [83]. SEC inhibitors, which presents a novel class of TMi’s, specifically inhibit MYC-target genes which is most effective in MYC-driven cancers [82]. In addition to transcriptional regulation, N-MYC and c-MYC link transcription-elongation to suppressing double strand breaks at active promoters, by recruiting BRCA1 (N-MYC), and by PAF1C loading (c-MYC) onto RNA polymerase II and altering chromatin structure via ubiquitin-mediated regulation [84,85,86]. Thus, while BRD4 and CDK7 particularly seem to influence super-enhancer linked *MYC* expression and subsequently MYC-target gene expression, CDK9 is mostly an effector of MYC. Given the dependency on the TM of (super-enhancer driven) transcription, and subsequent transcriptional amplification by MYC, these TMi’s seem to be an effective strategy to indirectly target MYC-driven cancers.

## 4. TP53 Loss in TNBC and Sensitivity to TMi’s

Although mutations in the *TP53* gene are present in almost half of human cancers, they are strongly enriched in TNBC (approximately 80% mutated and 55% copy number losses), making the loss of this tumor suppressor a key TNBC driver event [9]. TNBC frequently has a loss of one *TP53* allele, and a mutation in the other allele. While most mutations lead to a loss of function, other mutations can be dominant-negative and cause a gain of function, thereby inhibiting the wildtype (WT) p53 protein and apoptosis and promoting cell growth [87]. Because of the high abundancy of these mutations, restoring p53 activity and exploiting vulnerabilities of *TP53*-mutant cancers are attractive strategies for therapy. However, while therapies aiming to rescue p53 function are currently in clinical trials, these have not yet led to any major successes [88]. TMi’s are often associated with induction of p53-dependent responses, and p53 mutation status may therefore alter sensitivity to these drugs.

### 4.1. CDK9 Inhibition Overcomes Negative Regulation of p53 Stability

CDK9 inhibition particularly stabilizes WT p53 by disrupting the phosphorylation or expression of its negative regulators MDM2, MDM4, SIRT1 or iASPP [89,90,91,92]. Therefore, in this context, p53 WT cells are more sensitive to CDK9 inhibition than cells deficient in p53 [91]. Despite these p53-dependent effects, multiple studies report that p53-deficient cells are not more resistant to CDK9 inhibition as single treatment [78,93]. Combined inhibition of casein kinase 1α, CDK7 and CDK9 does cause a p53-dependent cell death by simultaneous induction of DNA damage and p53 restoration via MDM2 inhibition [92]. Alternatively, CDK9 can also promote p53-dependent pro-survival responses, which may promote DNA repair and therefore protect cells against various stressors [94]. This p53-dependent pro-survival transcription can be prevented by CDK9 inhibition, sensitizing cells to topoisomerase inhibitors or other genotoxic stressors [94,95]. Thus, CDK9 inhibition has pleotropic effects on p53 signaling, which may primarily have implications for combination therapy with therapies that act through p53.

### 4.2. BRD4 Interacts with (Mutant) p53 to Induce Gene Transcription

BRD4 can directly interact with p53 and recruit it to chromatin [96,97]. The effects of these interactions are not consistent, and may be model-dependent. For example, BRD4 inhibition was also associated with induction of p53 target genes, indicating a potential repressive function of BRD4 and causing synergy with MDM2 inhibitors [96]. Moreover, BRD4 inhibition reduced SIRT1 phosphorylation and (mutant) p53 acetylation and induced P21 expression and senescence [98]. BRD4 inhibitors may also specifically interfere with mutant p53, with gain of oncogenic functions, which would be a specific vulnerability in *TP53* gain-of-function mutant cancers, including TNBC. This mutant p53 shapes the enhancer landscape and induces the binding of BRD4 to enhancer regions in response to chronic immune signaling, promoting cancer growth [99,100]. BRD4 inhibition prevents BRD4 co-recruitment with the oncogenic mutant p53 and activation of its target genes, and negatively impacts the stability of this mutant p53 protein [98,99]. Overall, the effect of BRD4 inhibition on p53 may thus depend on its mutation status, although combination therapy with p53-inducing agents, such as MDM2 inhibitors in p53-deficient TNBC will likely not be effective. However, causal effects of mutant p53 or p53-deficiency on BET inhibition sensitivity are not yet reported.

### 4.3. CDK7, CDK8 and CDK12/13 Inhibitors and Induction of p53 Responses

CDK7 inhibition does not seem to be dependent on p53 status as it is highly efficient in p53-mutated or p53-deficient cell lines, including TNBC, although thorough comparisons are lacking [101]. However, p53 activation induced by the MDM2 inhibitor Nutlin and 5-FU increases dependency of p53-dependent transcription by CDK7. CDK7 inhibition shifts the balance from pro-survival to pro-apoptotic transcription of p53 targets [102]. This combination therapy is therefore not effective in p53-deficient cells. Moreover, CDK7 inhibition also reduced the expression of mutant p53 in TNBC cells, while upregulating WT p53 in ER+ breast cancer cells, although the exact mechanism underlying this potential selectivity remains elusive [103]. Similar to interactions with p53 and BRD4, CDK8 binds to p53 target genes and is a co-activator of the p53 transcriptional program in response to p53-activating stimuli [104]. Furthermore, cyclin K, the binding partner of CDK12, interacts with SETD1A. The knockout of *CDK12* or *CCNK* suppresses induction of DNA damage response genes, and subsequently induces DNA damage and p53-dependent apoptosis [105]. However, CDK12/13 inhibition may not solely induce apoptosis via p53-dependent pathways, as many studies describe high sensitivity to these inhibitors in p53-deficient cancer cells and this p53-dependency may be more relevant in specific hematologic cancers. In colorectal cancer, p53-deficiency is even correlated to sensitivity of CDK12/CCNK degradation [106].

### 4.4. Specific Vulnerability Due to Monoallelic P53 Loss Concomitant with POLR2A Loss

Despite the frequent loss of *TP53* in TNBC, this also presents a unique therapeutic vulnerability. The *POLR2A* gene is almost always hemizygously co-deleted with *TP53* in human cancers, which is the case for up to 53% of TNBC patients and is even more frequent in stage III TNBC patients [34,107,108]. This concomitant decrease in RNA polymerase II levels increases sensitivity to direct depletion or inhibition of RNA polymerase II. This selective sensitivity was further increased using α-amanitin-conjugated trastuzumab in cells expressing (low) levels of HER2 [107,108]. In addition, *POLR2A* siRNA more efficiently targeted TNBC cells carrying hemizygous losses of *TP53* and *POLR2A* [34].

Altogether, while the frequent p53-deficient background in TNBC often causes drug resistance, this does not seem to be a limitation when inhibiting either CDKs or BET proteins. However, a systematic comparison of p53-deficient versus p53-proficient background in responses to these inhibitors is often lacking, which may be critical as at least part of their effects may be mediated through p53. While BET, CDK7 and CDK9 inhibition can interfere with p53-induced transcriptional programs, the combination treatments of TMi’s with p53-inducing agents will not be effective in p53-deficient TNBC.

## 5. TMi’s Interfere with DNA Damage Repair and the Replication Machinery

**Figure 4:**
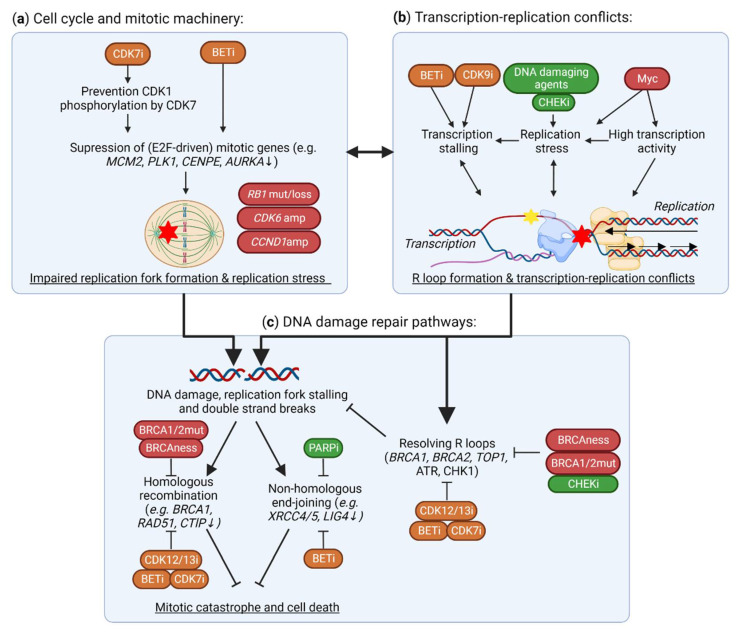
Effects of TMi’s on DNA damage and the DNA damage response. (**a**) CDK7 and BET inhibitors suppress the expression of mitotic genes (e.g., *MCM2, PLK1)*, and thereby functioning of the replication machinery, causing replication fork stalling, transcription-replication conflicts and DNA damage. In TNBC, *CDK6* and *CCND1* amplifications (amp), and *RB1* mutations (mut) or losses, cause further instability of the replication machinery. (**b**) In addition to this replication stress from improper functioning of the replication machinery, BET and CDK9 inhibitors cause stalling of the TM, thereby causing R loop formation, transcription-replication conflicts and, subsequently, DNA damage. In TNBC, these conflicts are further driven by *MYC* amplifications that drive high transcriptional activity and replication stress. (**c**) Although these R loops and DNA double strand breaks may be repaired through proper functioning of DNA damage response and repair, these pathways are impaired in homologous recombination deficient TNBC. TMi’s also suppress the expression of DNA damage response genes (e.g., *BRCA1*, *RAD51*), preventing DNA damage repair and thus inducing further DNA damage, all together leading to mitotic catastrophe and cell death. This figure was created with BioRender.com, accessed on 8 August 2022.

In addition to *TP53* loss, TNBCs often have other defects in DNA damage response and repair, including mostly defects in homologous recombination. Homologous recombination deficiency (HRD) has been identified in 50–70% of TNBC tumors, which is frequently caused by *BRCA1/BRCA2* mutations, copy number losses or promoter methylations. This HRD phenotype in sporadic tumors is also referred to as “BRCAness” [109,110,111] (Figure 1a,b). Although HRD is synthetic lethal with PARP inhibitors, the effect of PARP inhibitors on survival of TNBC patients has still been limited by multiple mechanisms of resistance, including restoration of functional BRCA1 expression, or rewiring of the DNA damage response. In addition to DNA damage response deficiencies, TNBCs often have genetic alterations of regulators of the cell cycle and mitosis, e.g., *RB1* losses or mutations and amplifications in *CCND1*/*CCNE1* and *CDK6*, which further drive cell cycle progression despite the high DNA damage burden (Figure 1a,b). TMi’s also induce DNA damage and specifically disrupt DNA damage repair mechanisms, including homologous recombination, and the replication machinery (Figure 4).

### 5.1. Suppression of DNA Damage Repair Genes by TMi’s

Many studies have demonstrated that CDK12 inhibition specifically affects the expression of long genes involved in homologous recombination and DNA replication (Figure 4c) [27,35,36,112,113,114]. CDK12 inhibition induces intronic polyadenylation, which is more prominent in these long intron-rich genes, potentially by suppressing phosphorylation of key pre-mRNA processing proteins [27,112]. Further selectivity of CDK12 for DNA damage response genes may also be mediated by SETD1A, that regulates recruitment of Cyclin K/CDK12 to these genes [105]. By reducing the expression of DNA damage response genes, CDK12 inhibition induces HRD, and this causes synergy with PARP inhibitors and cisplatin [36,115,116,117,118]. Even in HRD TNBC cells and PDX models, CDK12 inhibition reverses de novo and acquired PARP inhibitor resistance by further inhibiting residual or restored homologous recombination activity [118]. From a clinical perspective, biallelic loss-of-function mutations of *CDK12* are identified in cancer, and are relatively frequent in ovarian and prostate cancers [119,120]. These *CDK12*-mutant tumors have high genomic instability, evidenced by high frequency of tandem duplications. Although TNBC tumors are not associated with *CDK12* mutations, some TNBC tumors, especially those associated with *TP53* and *BRCA1* or *BRCA2* mutations, also display a large frequency of tandem duplications, albeit with a difference in size distribution compared to those observed for *CDK12*-mutated tumors [121].

BET inhibition also strongly disrupts the expression of DNA damage repair genes (Figure 4c), including homologous recombination genes *BRCA1* and *RAD51*, by preventing the association of BRD4 with their enhancers and promoters [122,123,124]. Moreover, it also suppresses genes involved in non-homologous end-joining, such as *XRCC4* and *XRCC5* [125,126,127]. Importantly, BRCA1 deficiency further sensitizes breast cancer cells to BRD4 inhibition, as this inhibition suppresses *MYC* expression and transcription and thereby induces oxidative stress and DNA damage [128]. Synergy of BET and PARP inhibitors has been demonstrated in various models, including TNBC, which is mostly mediated through BRD4 inhibition, but also partly through BRD2 and BRD3 [123,124,126,129,130]. Even in HRD models, BET inhibition still potentiates PARP inhibition, by preventing RAD51 and CtlP expression and overcoming multiple mechanisms of PARP inhibitor resistance [123,124,131]. Altogether, TNBCs with HRD may be just as vulnerable to BET inhibition alone or in combination with PARP inhibitors, and may be less capable of developing drug resistance. Moreover, BRD4 inhibition sensitizes cells to radiotherapy, ATR and CHK1 inhibition by attenuating various DNA repair mechanisms and synergistically increasing DNA damage [132,133,134]. Nevertheless, as DNA damage response genes are often not driven by super-enhancers, it remains unclear how preferentially BRD4 binds to these gene regions and how these genes are specifically vulnerable to BET inhibition.

CDK7 and CDK9 have also been shown to interfere with DNA damage responses. Genotoxic stress and p38 MAPK signaling activate CDK9, which promotes transcription of short pro-survival genes, such as *MCL1* and *CDKN1A* [94]. Disruption of this by CKD9 inhibitors therefore sensitizes cells to inducers of genotoxic stress, such as DNA damaging agents. Inhibition or depletion of CDK7 also disrupts expression of homologous recombination DNA damage response genes, including *BRCA1* and *RAD51*, and other DNA repair and cell division genes [29,135]. This subsequently sensitizes cells to PARP inhibitors or radiation. Altogether, CDK7, CDK12 and BRD4 are implicated in specifically regulating DNA damage response genes, and their inhibition induces HRD. These therapies often synergize with PARP inhibition, which may be a potent strategy in treating TNBC.

### 5.2. Aberrant Expression and Function of DNA Replication Machinery and Cell Cycle Genes

In addition to specific regulation of DNA damage genes, TMi’s can also suppress expression of genes involved in the DNA replication machinery (Figure 4a). BET inhibitors reduce expression of multiple DNA replication genes, including *AURKA*, *AURKB*, *CDK4* and *CCND1*, in multiple cancer types, including TNBC [136,137,138,139,140]. Thereby BETi’s sensitizes cancer cells, even in RB1-deficient cells, to CDK4/6 inhibitors, which have currently been approved for the treatment of hormone-positive breast cancers [136,137,138,139]. BET inhibitor resistance is associated with upregulation of cell cycle genes, including *AURKA* and *PLK1*, which induces a therapeutic vulnerability to PLK1 inhibitors [141]. Specific regulation of cell cycle genes may be regulated through co-recruitment of BRD4 with E2F1 on their promoter. On the contrary, CDK8 represses E2F1 activity, thereby stimulating the β-catenin pathway and stemness [61]. Selective CDK7 inhibition does not globally reduce RNA polymerase II phosphorylation, but rather reduces phosphorylation of CDK1 by CDK7 and consequently, E2F-driven, cell cycle-related gene expression [142,143]. Inhibition of E2F-driven transcription has also been observed after CDK7 inhibition due to its super-enhancer related gene repression [144]. Moreover, a therapeutic vulnerability to CDK7 inhibition is induced through kinome re-wiring upon CDK4/6 inhibition, which results in increased EGFR signaling [145]. In addition, TNBC tumors often have *CCND1* amplifications, and *CCND1* overexpression reduces global transcription activity and increases RNA polymerase II pausing, thereby sensitizing lymphoid tumor cells to inhibition of the TM via CDK7 or CDK9 [146]. In addition, CDK12 inhibition repressed expression of DNA replication genes [114]. Finally, *CDK12*-mutant associated tandem duplications in prostate cancer are enriched for duplication of cell cycle-related genes, such as *MCM7* and *CCND1*, compared to other tandem duplication phenotypes [120,147]. This could indicate that *CDK12*-mutant tumors are more dependent on the expression of these genes, or that these genes are duplicated due to transcription stalling and subsequent DNA damage around these genes.

### 5.3. BRD4 and CDK9 Inhibition Induce Transcription-Replication Conflicts

In addition to regulation of transcription of DNA damage response and replication genes specifically, the TM also directly interacts with DNA repair and replication machineries. RNA polymerase II may stall at a site of a DNA lesion, which can be recognized by various factors and induce transcription-coupled repair. However, DNA damage and prolonged RNA polymerase II stalling can also induce the annealing of the transcribed RNA strand with the template DNA strand, leaving the non-template DNA strand as single-stranded DNA, structures called R-loops [148]. R-loops more frequently occur with increased transcriptional activity [149,150]. If not properly resolved and repaired, they will lead to DNA damage and genomic instability through a variety of mechanisms, including transcription-replication conflicts, blocking the replication fork and inducing replication stress (Figure 4b) [148]. Ultimately, these structures can induce DNA damage, including double-strand breaks, and, eventually, mitotic catastrophe and cell death.

Importantly, TMi’s can induce R-loop formation and disrupt their repair (Figure 4b). BET inhibition deregulates transcription of BRD4-enriched genes, and thereby specifically induces R-loop formation on its target genes, leading to severe replication stress and DNA damage [151,152,153]. Similarly, transcription stalling by CDK9 inhibition also causes R-loop formation [154]. Various DNA damage repair proteins and DNA-RNA helicases, including BRCA1/2 and topoisomerase I, are involved in the prevention and resolving of R-loops [155]. These factors preventing and repairing R-loops are also disrupted by BET inhibition, for example by suppressing expression of topoisomerase II binding protein 1 and topoisomerase I [153,156]. These associations with R-loop formation are primarily described for CDK9 and BRD4 inhibition, but may also be important upon CDK12 inhibition, as this also interferes with the expression of related genes and induces DNA damage. Altogether, TMi’s can induce DNA damage by stalling transcription in general, and by specifically interfering with the transcription of DNA damage response and cell cycle genes.

## 6. Targeting the TM to Unleash Anti-Cancer Immunity against the High Mutational Burden in TNBC

**Figure 5:**
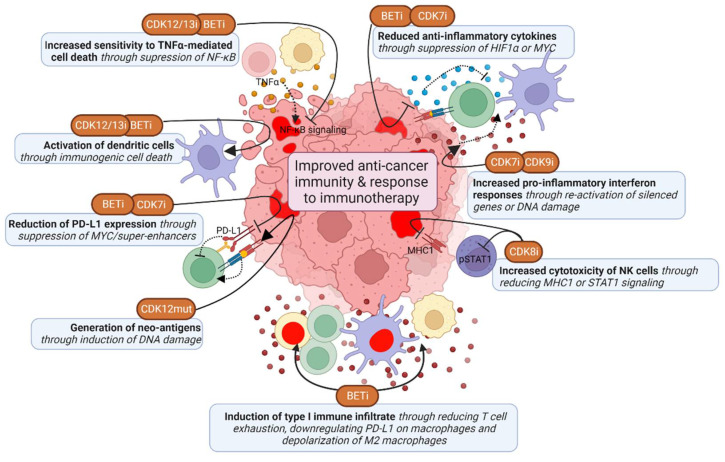
Effects of TMi’s on anti-cancer immunity. TMi’s improve anti-cancer immunity by suppressing expression of anti-inflammatory cytokines and PD-L1 in cancer cells, but also in immune cells directly, thereby inducing inflammatory type 1 immune responses. TMi’s also induce pro-inflammatory IFN-y responses in cancer cells, induce sensitivity to TNFα-mediated cell death and induce immunogenic cell death. CDK8 inhibition mostly improves anti-cancer immunity by enhancing natural killer (NK) cell cytotoxicity. This figure was created with BioRender.com (accessed on 8 August 2022).

Given the high frequency of deficiencies in homologous DNA recombination and p53 in TNBC, these tumors have a higher mutation and neo-antigen burden, and may therefore be more immunogenic than other breast cancer tumors [157]. Some TNBC tumors also have high T cell infiltrates, and expression of immunosuppressive PD-L1 and PD-L2 is enriched in high-risk triple-negative breast cancer [158,159]. PD-L1 inhibitors atezolizumab and pembrolizumab have been FDA approved for the treatment of PD-L1-positive TNBC, which does significantly improve overall and progression-free survival of TNBC patients [160,161]. However, despite this significant improvement, this therapy is not effective in all patients, and there is thus a need to further improve its efficacy. TMi’s may improve anti-cancer immunity and thereby enhance the effect of these therapies (Figure 5).

### 6.1. CDK12 Deficiencies Increase Fusion Neoantigens and Immunogenicity

As mentioned earlier, *CDK12* loss-of-function mutant tumors have a distinct genetic phenotype, characterized by large tandem duplications [120]. These tandem duplications lead to gene fusions, which subsequently cause open-reading frames and a high neoantigen burden [30,120]. These mutant tumors also have elevated expression of chemokine signaling genes associated with higher T cell infiltration scores [30,120]. Accordingly, chemotherapy-naïve prostate cancer patients with biallelic *CDK12* mutations benefit from PD-L1 checkpoint inhibitors [120,162]. However, pharmacological CDK12 inhibition or depletion has not yet been linked to the generation of neoantigens. CDK12/13 inhibition does cause immunogenic cell death by inducing ER stress, thereby enhancing anti-PD-L1 therapy efficacy, dendritic cell and T cell activation and infiltration [163]. Moreover, CDK12 is required for expression of target genes from the inflammatory NF-κB signaling pathway, and CDK12 inhibition increases sensitivity to TNFα-induced cell death [164,165]. Altogether, *CDK12* mutations and inhibition may thus induce immunogenicity of tumors and potentiate immunotherapy.

### 6.2. Induction of Interferon Responses by CDK7 and CDK9 Inhibition

While complete CDK9 inhibition shuts down most (highly) active transcriptional programs, it also causes chromatin remodeling and thereby re-activation of epigenetically silenced genes [166]. These genes include endogenous retroviral elements, which provide tumor-specific T cell epitopes and induce antiviral IFN-γ responses, including the upregulation of HLA molecules [166]. Consequently, CDK9 inhibition induces the infiltration of T cells and activates dendritic cells into the tumor environment, which is further potentiated by PD-L1 inhibition. CDK9 also directly interacts with YY1, a regulator of the 3D chromatin structure. Inhibition of this complex also induces interferon responses and regulatory T cell reduction, leading to sensitization to anti-PD-L1 therapy [167]. Corresponding to this, high CDK9 levels are associated with tumor stage and lower CD8+ T cell infiltration and increased T cell exhaustion [168]. However, the first generation CDK9 inhibitors that are not fully selective for CDK9, have been associated with reduction of pro-inflammatory signaling within tumors and reduced T cell activation [169,170]. These effects may be due to non-selective CDK inhibition, prompting for the use of highly selective inhibitors. Altogether, specific CDK9 inhibition seems to have beneficial effects on the immune response, particularly via interferon responses. However, caution is needed for evaluation of potentially negative effects on cancer immunity.

CDK7 inhibition also induces IFN-γ and TNFα signaling responses, which is caused by DNA damage and micronuclei formation rather than chromatin remodeling [143]. Subsequently, CDK7 inhibition increases tumor infiltration of activated effector CD4+ T cells and cytotoxic CD8+ T cells, enhancing combination treatment with anti-PD-1 antibodies. Furthermore, CDK7 inhibition also enhances anti-PD-1 therapy by suppressing expression of MYC target genes, including PD-L1, and thereby induced infiltration of CD8+ T cells [171]. CDK7 inhibition also prevents the upregulation of immunosuppressive genes upon EGFR CAR T-cell therapy [172]. Although CDK7 inhibition also suppresses the expression of inflammatory genes associated with super-enhancers in activated macrophages, this is a beneficial effect in combination with CAR T cells, preventing cytokine release syndrome [173].

### 6.3. BRD4 Inhibition Suppresses Immune Escape Mechanisms

BET inhibitors are associated with promoting pro-inflammatory type 1 immune subsets, while suppressing pro-tumor type 2 immune subsets. In cancer cells BRD4 inhibition suppresses super-enhancer and IFN-γ driven transcription of PD-L1 (*CD274* gene) by preventing binding of BRD4 and IRF1 to its promoter and enhancer regions [174,175,176,177,178]. BRD4 inhibition was also shown to decrease M2 tumor-associated macrophage (TAM) proliferation, particularly by directly inhibiting HIF1α expression in tumor cells, thereby inhibiting the secretion of M2-promoting colony-stimulating factor 1 (CSF1) [179]. Furthermore, BRD4 inhibition induces the immunogenic cell death of cancer cells and subsequently induces phagocytosis by dendritic cells and expansion of CD8+ cytotoxic T cells [180,181,182]. BET inhibition also interferes with NFκB signaling by preventing recruitment of BRD4 to NFκB target genes, and thereby sensitizes cells to TNFα-induced cell death and to T cell or immune-checkpoint blockade therapy in a TNFα-dependent manner [183,184]. BET inhibitors also have a direct effect on immune cells. BRD4 inhibition has been demonstrated to directly improve anti-cancer (CAR) CD4+ and CD8+ T cell activity, for example by inducing cytotoxicity and reducing T cell exhaustion [185,186,187,188]. BRD4 inhibition also suppresses IFN-γ responses of T cells, which prevents PD-L1 upregulation of TNBC cells [189]. In addition, BET inhibitors reduce PD-L1 levels on tumor-associated dendritic cells and macrophages, and thereby increase cytotoxic activity of tumor-associated T cells [174,190]. Moreover, BRD4 inhibition promotes depolarization of M2 macrophages to M1 macrophages, reduces regulatory T cells and enhance NK-cell mediated toxicity [191,192,193]. Alternatively, BRD4 inhibition might also inhibit immune surveillance, for example of oncogene-induced senescent cells during tumor development by preventing secretion of inflammatory cytokines [194]. The described inhibitory effects on IFN-γ responses can also reduce immune responses, which is, however, not frequently described in the context of BET inhibitors. Thus, while BET inhibition primarily seems to have positive effects on anti-cancer immunity, their potential negative effects do also need further investigation.

### 6.4. CDK8 Inhibition Potentiates Natural Killer (NK) Cell Activity through STAT1 Inhibition

The effects of CDK8 inhibition on anti-cancer immunity described so far are mostly limited to promoting anti-cancer NK cell activity [195,196,197]. These effects may partly be mediated via inhibition of CDK8-dependent STAT1 (S727 residue) phosphorylation in NK cells directly, which enhanced their cytotoxic activity. Although CDK8 regulation of pause-release via STAT1 is required for IFN-γ-induced cytotoxic activity of NK cells, NK cells can also be activated through other stimuli, such as IL-12, enabling CDK8-independent activation [198,199]. CDK8 inhibition or knockdown also inhibits IFN-γ-induced STAT1 phosphorylation in TNBC cells [196]. This causes induction of ICAM-1 and reduction of MHC1 and PD-L1, which both may otherwise inhibit NK cells, and thereby induces NK-cell mediated tumor cell clearance. In summary, TMi’s can improve anti-cancer immunity mostly by inducing pro-inflammatory responses and suppressing anti-inflammatory responses in both immune and cancer cells.

## 7. TMi’s Cooperate with Inhibitors of Growth Factor or Hormone Signaling Pathways

Genes driving and/or controlling MAPK and PI3K signaling pathways are often altered in TNBC tumors. For example, TNBC tumors often have *PTEN* or *NF1* losses or mutations, which normally repress the PI3K or PI3K/MAPK pathways, respectively (Figure 1). Moreover, TNBC and other breast cancer subtypes have a high frequency of *AKT3* amplifications, *PIK3CA* amplifications and mutations [8,9]. TNBCs also occasionally have *EGFR*, *BRAF* or *KRAS* amplifications, gains, or mutations. While inhibitors of these pathways have not resulted in improved clinical responses, TMi’s may prevent the development of drug resistance to these inhibitors (Figure 2).

### 7.1. Preventing Kinome Reprogramming upon MAPK/PI3K Pathway Inhibition Using BET Inhibitors

Although BET inhibition is not usually associated with reduced transcription of components of the MAPK pathway specifically, it may particularly prevent drug resistance to MEK inhibitors. Multiple studies have demonstrated that combined BRD4 and MEK inhibition is beneficial, especially in KRAS/NRAS-driven or NF1-deficient cancer, including TNBC [200,201,202,203,204,205]. Mechanistically, MEK inhibitor resistance is often associated with kinome reprogramming. This causes upregulation of MYC or of various receptor tyrosine kinases, and which subsequently stimulate MAPK and PI3K signaling [204,205,206,207]. This kinome reprogramming can be mediated through enhancer remodeling and increased binding of BRD4 to those enhancers or promoter regions [204,205]. BRD4 and CDK9 depletion or inhibition prevents this upregulation and BRD4 inhibition disturbs the global enhancer remodeling, sensitizing TNBC to MEK inhibition [204,205,207]. Elevated RAS/MAPK activity also causes resistance to BET inhibitors, which can be caused by a downregulation of negative regulators of the MAPK pathway after BRD4 inhibition [203,208,209]. Therefore, MEK inhibition also sensitizes cells to BET inhibitors.

BET inhibition similarly sensitizes cells to PI3K pathway and EGFR inhibition. Resistance to Akt and PI3K inhibition is also mediated through chromatin remodeling and increased binding of multiple transcription factors, including BRD4, to gene regions of *MYC* and multiple receptor tyrosine kinases [210,211]. BRD4 inhibition reverses this mechanism of drug resistance and sensitizes cells to PI3K or Akt inhibition. Importantly, *PIK3CA* mutations confer resistance to BRD4 inhibition in breast cancer, which can be reversed using PIK3CA-specific or mTOR inhibitors [212]. *PI3KCA* mutations or *PTEN* losses also cooperate with *MYC* overexpression in mammary tumorigenesis [213]. Although PTEN directly regulates the activity of PI3K, it also suppresses transcription in TNBC through PTEN binding to chromatin and interaction with RNA polymerase II, CDK7 and CDK9 [214]. Thereby, PTEN deficiency is associated with sensitivity to TMi’s.

Although functional associations between these PI3K/MAPK pathways and transcriptional CDKs have also been identified, the synergistic potential of these combinations are not widely described [215]. While many studies describe synergy with CDK7/12/13 inhibitors THZ1, THZ531 and inhibitors of receptor tyrosine kinases and MAPK or PI3K signaling, these mechanisms are often inconsistent or lacking. The potency of these CDK inhibitors, including THZ1 and THZ531, is strongly influenced by ABCG2, and many tyrosine kinase inhibitors, including EGFR inhibitors can inhibit ABCG2, which clouds the mechanism behind this synergistic action [216,217]. Nevertheless, given the collaboration of p-TEFb and BRD4, and the similar binding of BRD4 and CDK7 to active enhancers, inhibitors of CDK7 and CDK9 may also sensitize cells to PI3K and MAPK pathway inhibitors.

### 7.2. Targeting Androgen Receptor-Driven Transcription of Luminal TNBC

The luminal subtype of TNBC has a distinct genomic and transcriptomic landscape, associated with increased amplifications of *EGFR* and mutations in *PIK3CA*, and is more dependent on androgen receptor (AR) signaling [9]. AR is a nuclear hormone receptor and transcription factor, and like BRD4 it binds to active enhancers, and is more enriched at super-enhancers. Deregulation of AR signaling is also associated with chromatin relaxation, which is mediated through bromodomain-containing proteins including BRD4 [218]. While AR seems to influence BRD4 recruitment, BRD4 inhibition disrupts AR recruitment and AR-mediated gene transcription [41,218,219]. BRD4 inhibition is thereby more effective in cancer cells with high AR activity and dependency. Increased androgen-independent, but AR-dependent, super-enhancer activity can also drive resistance to antiandrogens and is dependent on BRD4 [220]. Besides these interactions with BRD4, both CDK7 and CDK9 phosphorylate and stabilize AR [221,222,223]. CDK9 inhibition thereby preferentially inhibits AR-driven oncogenic programs, especially of highly transcribed genes with high mRNA turnover [221]. Given these distinct mechanisms of AR regulation, BET inhibitor resistant prostate cancers demonstrated reactivation of AR signaling via CDK9-mediated phosphorylation of AR [224]. This induces a therapeutic vulnerability for CDK9 inhibitors. Antiandrogen therapy resistance is associated with increased MED1 phosphorylation, which can be prevented by CDK7 inhibition through inhibition of AR and MED1 recruitment to super-enhancers [225]. Although these studies generally focus on prostate cancer, which is mostly driven by AR signaling, these strategies, including BET inhibition can, for the same reason, also be especially effective in AR-driven TNBC [226]. Overall, TMi’s, and especially BET inhibitors, can overcome drug resistance to inhibitors of the MAPK and PI3K pathways by preventing adaptive transcription and TMi’s can target AR-dependencies.

## 8. Conclusions and Future Perspectives

Although the TM, including targetable transcriptional CDKs and BRD4, globally drives gene transcription, it is also an accomplice of the oncogenic (epi)genomic landscape in TNBC. TMi’s may specifically disturb this controlled network of oncogenic aberrations at its core. Given the large interpatient and intratumor heterogeneity and the lack of effective therapy, this strategy may be especially attractive in TNBC. Briefly, most of these inhibitors can induce DNA damage and interfere with its repair, suppress transcription of (super-enhancer driven) oncogenes, suppress transcriptional plasticity at the source of drug resistance, interfere with the function of oncogenic transcription factors and improve anti-cancer immunity (Figure 2). Although not discussed in this review, other cancer hallmarks such as cancer stemness and metabolism, can also be influenced by these inhibitors. While not discussed in much detail here, CDK8/19 inhibition does interfere with metabolic responses to hypoxia and reduces cancer stem cell properties [227]. Inhibition of the TM is a therapeutic strategy to indirectly target multiple important cancer hallmarks simultaneously, which potentially limits the development of acquired or intrinsic drug resistance.

Most genomic alterations in TNBC, such as *TP53* mutations, HRD and *MYC* amplifications do not seem to limit the effects of these inhibitors and rather induce therapeutic vulnerabilities. Overexpression of oncogenic transcription factors such as MYC, may even sensitize TNBC to these inhibitors. Nevertheless, mutations and copy number alterations in the MAPK/PI3K signaling pathways may limit their sensitivity, prompting for a combination treatment with inhibitors of these pathways. In contrast, the direct effect of genetic alterations in TNBC on the sensitivity to TMi’s is not sufficiently systematically studied. The effects by these alterations may be clouded by the large variety of different mutations and copy number variations present, which may all have different effects on sensitivity to these inhibitors. Moreover, the TM seems to be strongly exploited at highly active (onco)genes which are regulated by epigenetic factors, such as super-enhancers and histone modifications. While TNBC models specifically representing certain cancer mutations or copy number variations are available, systematic models for these epigenetic alterations are lacking. Moreover, while many studies have thoroughly investigated the genomic, transcriptomic, and proteomic landscape in TNBC tumors, the epigenomic landscape in human TNBC tumors, including super-enhancer driven gene transcription, for example of *MYC*, is relatively underexplored. Further knowledge of the epigenetic landscape in TNBC and its effect on sensitivity to TMi’s may be critical to understand the potential benefits of, specifically, CDK7, CDK8 and BRD4 inhibition, and therefore to guide rationally designed combination therapies and clinical trials.

Currently ongoing trials will point out the potential of these TMi’s. Theoretically, epigenetic and transcriptional inhibitors hold exciting promise for the future, especially for targeting heterogeneous diseases without uniform drivers, such as TNBC. The large amount of preclinical in vivo studies demonstrates proof of concept of their safety and efficacy. Understanding how the (epi)genomic landscape contributes to differential responses may be necessary to improve clinical outcomes and design effective combination therapies. This review discussed our current understanding of this with regards to the genomic alterations in TNBC. TMi’s induce various therapeutic vulnerabilities, such as sensitivity to DNA damaging agents and PARP inhibitors, or sensitivity to immunotherapy such as immune checkpoint inhibitors, which have recently been approved as new therapies for TNBC. Although the mechanisms behind the specific regulation of gene transcription by the TM is not fully understood, this gene-specific regulation provides therapeutic opportunities for targeting multiple heterogeneous TNBC drivers at once.

## Figures and Tables

**Table 1:** Overview inhibitors of the TM and their proof-of-concept in TNBC in vivo studies.

Target	Selection Most Selective Inhibitors ^a^	Tested in TNBC In Vivo (*PMID*) ^b^	Proposed Specific Effect In Vivo Model ^c^	Combination Therapy or Formulation ^c^
**RNA pol II**	α-amanitin, Oncrasin-1	α-amanitin (*33568521*)	Inhibition of global transcription	Formulated as HER2-conjugated drug
**CDK7**	BS-181, THZ1, THZ2, LDC4297, LY3405105, ICEC0942 (Samuraciclib), IV-361, YKL-5-124, SY-1365 (Mevociclib)	THZ1/THZ2 (*26406377*),	Suppression of super-enhancer gene transcription	-
		THZ1 (*33875483*)	Suppression of immunosuppressive genes	EGFR CAR T cells
**CDK8/** **CDK19**	AS2863619, BI-1347, BRD6989, CCT251545, Corticostatin A, MSC2530818, SEL120, Senexin A/B, JH-XI-10-02 (CDK8i only), JH-XVI-178	BI-1347 (*32024684*)	Activation of NK cells	SMAC mimetic
		CDK8 shRNA (*34689158*)	Suppressing metastatic genes and activation of NK cells, effect on re-growth and metastasis	-
**CDK9**	NVP2, AZ5576, LDC000067, AZD4573, BAY-1143572, BAY-1251152 (VIP152), LY2857785, (THAL-)SNS-032	Compound 45 (*34538051*)	Downregulation key oncogenes, e.g., MYC and Mcl-1	-
		Complex 1 (*35530158*)	Especially anti-metastatic, also MYC/Mcl-1 levels reduced	-
		NVP2 (+CDK1i, *33417832*)	Inhibition of persister cells with reduced MYC expression	Docetaxel
		4ab (+CDK2i, *29144137*)	Not described	-
**CDK12/** **CDK13**	THZ531, SR4835, BSJ-4-116 (CDK12i only)	SR4835 (*31668947*)	Suppression DNA damage response genes	DNA damaging agent & PARP inhibition
		SR4835 (*32941949*)	Immunogenic cell death	Anti-PD1 therapy
**BET/BRD4**	A1874, ABBV-075 (Mivebresib), ARV-771, ARV-825, BI-2536, Bromosporine, CPI-0610 (Pelabresib), CPI-203, dBET6, I-BET151 (GSK1210151A), I-BET726 (GSK1324726A), JQ1, MS417, MS436, MZ1, OTX015 (Birabresib), PF-6405761 (PFI-1)	JQ1 (*27292261*)	Suppression of hypoxia-induced genes and angiogenesis	-
		JQ1 (*32735909*)	Expression of mitotic genes confers resistance to BETi	PLK inhibition
		JQ1 (*27650498*)	Suppression of aurora kinases	-
		JQ1 (*26735014*)	Basal to luminal dedifferentiation	-
		JQ1 (*32339606*)	Suppression of PD-L1 expression induced by IFN-y	-
		JQ1 & MS417 (*24525235*)	Suppression WNT5a expression and stem cell properties	-
		JQ1 & dBET-6 (*32416067*)	Multiple factors synthetically lethal in JQ-1 resistant cells	JAK2, BCL2/BCL-XL, CDK4/6 inhibition
		JQ1 & INCB054329 (*32161105*)	Suppression of N-MYC	MEK inhibition
		BETd-246 (*28209615*)	Suppression of Mcl-1	BCL-XL inhibition
		MZ1 (*31470872*)	NA (G2/M arrest and apoptosis)	-
		i-BET151 (*28108460*)	Suppression of chromatin remodeling upon MEKi	MEK inhibition
		OTX-015 pro-drug (*33739832*)	Improved pharmacokinetics and reduced toxicity	Bottlebrush pro-drug formulation

^a^ Selection of commercially available, selective inhibitors. ^b^ Publicly available in vivo studies in TNBC, excluding pan-CDK inhibitors with PubMed IDs (PMID) in italic. ^c^ Corresponding to study in same line of previous column.

**Table 2:** Overview of clinically tested TMi’s.

	Inhibitors ^a^	Cancer Type ^b^	Mono- or Combination Therapy	Trial ID, Phase and Status ^c^
**CDK7i**	SY5609	HR+ BC, SCLC, PanCa	Fluvestrant, Gemcitabine or Nab-paclitaxel	NCT04247126 (Phase I)
	SY1365	OvCa, HR+ BC	Carboplatin, Fulvestrant	NCT03134638 (Phase I, terminated due to management decision)
	XL102	OvCa, TNBC, HR+ BC, CRPC	Mono (TNBC) or Fulvestrant, Abiraterone, Prednisone	NCT04726332 (Phase I)
	CT7001	TNBC, CRPC, HR+ breast cancer	Mono (TNBC) or Fulvestrant	NCT03363893 + NCT04802759 (Phase I/II)
**CDK9i**	AZD4573	Hematologic	Mono	NCT03263637 (Completed, Phase I), NCT05140382 + NCT04630756 (Phase I)
	PRT2527	Sarcomas, CRPC, HR+ BC, TNBC, tumors with *MYC* amplification	Mono	NCT05159518 (Phase I)
	GFH009	Hematologic	Mono	NCT04588922 (Phase I)
	Fadraciclib/CYC065	Various solid and lymphoma, including TNBC	Mono or Venetoclax	NCT02552953+ NCT05168904+ NCT04017546 (Phase I), NCT04983810 (Phase I solid tumors, Phase II lymphoma)
	KB-0742	Solid tumors and non-Hodgkin lymphoma	Mono	NCT04718675 (Phase I)
	BAY 1251152	Solid and hematologic	Mono or Pembrolizumab	NCT04978779+ NCT02635672 (Phase I), NCT02745743 (Phase I, completed)
	BAY1143572	Various solid and hematologic	Mono or G-CSF	NCT01938638+ NCT02345382 (Phase I, completed)
	TP-1287	Solid tumors and sarcoma	Mono	NCT03604783 (Phase I)
**CDK8i**	TSN084 (also other targets)	Various solid and hematologic	Mono	NCT05300438 (Phase I)
	SEL120	AML, myelodysplastic syndrome	Mono	NCT04021368 (Phase I)
	BCD-115	ER+HER2- BC	Endocrine therapy	NCT03065010 (Phase I, completed)
**BET/BRD4i**	ZEN-3694	Solid/lymphomas	Talazoparib, Ipilimumab/Nivolumab, Enzalutamide/Pembrolizumab, Talazoparib, Binimetinib, Entinostat, Etoposide+Cisplatin	NCT05327010 + NCT04471974 + NCT05071937 (Phase II), NCT04840589 + NCT05111561 (Phase I) NCT05053971 + NCT05019716 (Phase I/II), NCT02711956 (Phase I/II, completed), NCT02705469 (Phase I, completed)
	FT-1101	Hematologic	Azacitidine	NCT02543879 (Phase I, completed)
	RO6870810	Multiple myeloma	Daratumumab	NCT02543879 (Phase I, completed)
	TQB3617	Malignant tumors	Mono	NCT05110807 (Phase I)
	CPI-0610	Multiple myeloma, lymphoma	Mono	NCT02157636 + NCT01949883 (Phase I, completed, NCT02158858 (Phase 1/2)
	BMS-986158 or BMS-986378	Pedriatric solid tumors/brain tumors and lymphoma	Mono or Ruxolointib	NCT03936465 (Phase I)
	BMS-986158	Various solid and hematologic	Mono or Nivolumab	NCT02419417 (phase I/II)
	GSK525762	Various hematologic/solid, incl. TNBC	Mono	NCT01943851 (Phase II, completed), NCT01587703 (Phase I, completed)
	NUV868	Solid cancers	Mono or Olaparib, Enzalutamide	NCT05252390 (Phase I/II)
	INCB054329	Various solid/hematologic	Mono	NCT02431260 (Phase 1/2: Terminated due to PK variability)
	CC-95775	Solid, non-Hodgkin lymphomas	Mono	NCT04089527 (Completed, Phase I)
	OTX015	NUT-midline carcinoma, TNBC, NSCLC, CRPC, PDAC, AML, Lymphoma	Mono	NCT02259114 + NCT01713582 (Phase I, completed), NCT02698176 + NCT02698189 (Phase I, terminated: limited efficacy), NCT02296476 (Phase II, terminated: lack of activity)
	CC-90010	Solid, non-Hodgkin lymphomas	Mono	NCT03220347 (Phase I)

^a^ Relatively selective inhibitors that are currently, or have been, in clinical trials according to clinicaltrials.gov for the treatment of various malignancies, excluding withdrawn studies and clinical trials for other diseases. ^b^ Abbreviations: OvCa, ovarian cancer; PanCa, pancreatic cancer; CRPC, castration-resistant prostate cancer; HR+ BC, hormone receptor positive breast cancer; (N)SCLC, (non-) small-cell lung cancer; PDAC, pancreatic ductal adenocarcinoma; AML, acute myeloid leukemia. ^c^ Information derived from clinicaltrials.gov (accessed on 27 May 2022).
